# Effects of Baduanjin exercise on cognitive impairment in older adults: a systematic review and meta-analysis

**DOI:** 10.3389/fpubh.2025.1586011

**Published:** 2025-07-03

**Authors:** Xiao-Gang Gong, Le-Peng Wang, Ling-Ling Yang, Feng Liu, Dao-Ning Zhang, A-Yuan Zhang

**Affiliations:** ^1^Department of Medicine, College of Special Education, Beijing Union University, Beijing, China; ^2^School of Humanities, Beijing University of Chinese Medicine, Beijing, China; ^3^Henan Provincial Hospital of Traditional Chinese Medicine, Zhengzhou, China; ^4^Department of Diagnostics of Traditional Chinese Medicine, School of Traditional Chinese Medicine, Beijing University of Chinese Medicine, Beijing, China; ^5^Institute of Taiwan Studies, Beijing Union University, Beijing, China; ^6^College of Education, Capital Normal University, Beijing, China

**Keywords:** Baduanjin exercise, cognitive impairment, older adults, systematic review, meta-analysis

## Abstract

**Background:**

Cognitive impairment in older adults poses a growing burden on global healthcare systems, especially in resource-limited communities. Baduanjin exercise, a low-cost traditional Chinese mind–body exercise, demonstrates considerable potential for assisting older adults in managing cognitive impairment. However, there is no consensus regarding its efficacy. This meta-analysis aimed to assess the feasibility and effectiveness of Baduanjin exercise in ameliorating cognitive impairment in older adults.

**Methods:**

Randomized controlled trials (RCTs) published through January 30, 2025, were searched in PubMed, Web of Science, Cochrane Library, Embase, ClinicalTrials.gov, and Chinese databases (CSTJ, CNKI, Wanfang). Two reviewers independently conducted trial selection, assessed methodological quality using the Cochrane Risk of Bias tool, and extracted data in accordance with PRISMA guidelines. Seven RCTs involving 539 community-dwelling older adults met the inclusion criteria.

**Results:**

Baduanjin exercise significantly improved global cognitive function [mean difference (MD) = 2.15; 95% CI, 1.53 to 2.76; *p <* 0.00001], memory (standardized mean difference = 0.59; 95% CI, 0.38 to 0.80; *p <* 0.00001), executive function (SMD = 0.26; 95% CI, 0.07 to 0.44; *p =* 0.007), and physical health (MD = −0.86; 95% CI, −0.26 to −0.46; *p <* 0.00001). No included study reported adverse effects related to Baduanjin exercise.

**Conclusion:**

These findings indicate that Baduanjin exercise can effectively improve cognitive impairment in older adults. Nevertheless, further rigorously designed RCTs are required to confirm these findings.

**Systematic review registration:**

International Platform of Registered Systematic Review and Meta-Analysis Protocols (INPLASY) under the registration number INPLASY202460007, https://inplasy.com/inplasy-2024-6-0007/.

## 1 Introduction

The global population is aging rapidly. In 2020, one billion people were aged ≥ 60 years. This number is projected to increase to 1.4 billion by 2030, meaning one in six people worldwide will be in this age group. By 2050, the population aged ≥ 60 years is expected to double, reaching 2.1 billion (1).

With increasing life expectancy, cognitive impairment, which predominantly affects older adults, has emerged as a significant public health issue affecting tens of millions of individuals globally. This condition causes considerable distress to both patients and caregivers and imposes a financial burden on families and healthcare systems (2, 3). According to the World Health Organization, over 55 million people worldwide suffer from dementia, and nearly 10 million new cases are reported annually (4). Notably, an increasing number of people suffer from mild cognitive impairment (MCI) and cognitive frailty (CF).

Mild cognitive impairment (MCI) is a neurodegenerative condition characterized by a decline in memory, attention, logical thinking, and executive functions, and is defined as a precursor to dementia (5). The prevalence of MCI among older adults is estimated to range from 6.7 to 25.2% (6). Considering the exceptionally high risk of dementia in this clinical cohort, it is imperative to prioritize effective interventions aimed at mitigating further cognitive decline in older adults with MCI (7). CF is a relatively new clinical syndrome characterized by a combination of physical frailty and cognitive impairment, excluding dementia (8). A diagnosis of CF is determined when an individual exhibits both cognitive decline (i.e., memory or executive function impairment) and physical frailty (evidenced by slow walking speed, muscle weakness, or weight loss). The prevalence of CF among older adults ranges from 10.3 to 42.8% (9) and the incidences of MCI and CF tend to increase with age. MCI and CF increase the risk of developing Alzheimer’s disease, neurocognitive disorders, and vascular dementia in older adults and are associated with adverse health outcomes, such as functional disability, reduced quality of life, and increased mortality. However, the degree of cognitive impairment in conditions such as MCI and CF is potentially reversible. Early intervention may delay or even reverse the progression of these conditions, thereby reducing the incidence of associated adverse events (10). Despite the availability of various pharmacological treatments, such as cholinesterase inhibitors and NMDA receptor antagonists, their effectiveness is often limited and may entail adverse side effects (11). Therefore, exploring safe and effective non-pharmacological interventions is crucial.

Baduanjin, also known as the Eight Brocades, is a traditional Chinese qigong and mind–body exercise with a history of over a thousand years. Originating in ancient China, it has been passed down through generations as an important part of Chinese medicine and healthcare. Rooted in traditional Chinese philosophy and medicine, Baduanjin exercise is based on the concept of balancing the body’s energy (qi) and promoting the harmony of the mind and body (12). Among the various versions of Baduanjin exercise throughout its history, the version developed by the General Administration of Sports in China is currently the most popular. This exercise consists of eight simple movements combined with deep breathing and mental focus, allowing completion in a relatively short time. Unlike conventional aerobic or resistance exercises that primarily focus on strengthening the body, Baduanjin exercise emphasizes the integration of body posture, deliberate movements, focused attention, deep breathing, and relaxation. This holistic approach aims to maximize both physical and mental well-being. Baduanjin exercise has recently received increasing attention, owing to its simplicity, safety, and comprehensive regulatory effects on various body systems (12). Regular Baduanjin exercise can enhance flexibility and balance (13, 14), improve cardiopulmonary function (15, 16), improve sleep quality (17, 18), and regulate emotions (12, 19), all of which can positively impact cognitive function.

Although mind–body exercises, such as Tai Chi and Yoga, have been extensively studied for cognitive benefits in older adults (20, 21), evidence for Baduanjin exercise remains fragmented. Existing meta-analyses either focus on mixed populations (e.g., middle-aged adults and patients with non-age-related cognitive decline) or combine Baduanjin exercise with other interventions (22, 23), limiting their applicability to community-dwelling older adults. To our knowledge, this is the first systematic review and meta-analysis exclusively evaluating Baduanjin exercise as a standalone intervention for older adults with MCI or CF, excluding confounding populations (e.g., stroke survivors or Alzheimer’s patients). By synthesizing RCTs and analyzing domain-specific cognitive outcomes (global cognition, memory, executive function), this study provides targeted evidence for integrating Baduanjin exercise into age-friendly public health strategies while preserving its traditional holistic principles.

## 2 Methods

### 2.1 Study registration

This systematic review and meta-analysis was registered on the International Platform of Registered Systematic Review and Meta-Analysis Protocols (INPLASY) under the registration number INPLASY202460007. This study was designed and implemented in accordance with the Preferred Reporting Items for Systematic Reviews and Meta-Analyses (PRISMA) guidelines (24).

### 2.2 Search strategy

A comprehensive search for eligible trial reports was conducted across several online databases: PubMed, Web of Science, Cochrane Library, Embase, ClinicalTrials.gov, China National Knowledge Infrastructure, Wanfang Knowledge Database, and Chinese Science and Technique Journal Database. The search concluded on January 30, 2025. To ensure the comprehensiveness and accuracy of the search, we combined subject headings and free-text terms in all databases. The primary keywords used in the initial search were “Baduanjin,” “cognitive,” “cognitive Impairment,” and “randomized controlled trial.” Subsequently, medical subject headings and thesaurus terms were incorporated to refine the search terms. For example, in PubMed, we used the following search strategy: (“Baduanjin” [Title/Abstract] OR “Ba duan jin” [Title/Abstract] OR “eight brocade” [Title/Abstract] OR “eight section brocades” [Title/Abstract]) AND (“Cognitive Dysfunctions” [Title/Abstract] OR “Cognitive Impairments” [Title/Abstract] OR “Cognitive Impairment” [Title/Abstract] OR “Cognitive Disorder” [Title/Abstract] OR “Cognitive Decline” [Title/Abstract] OR “Mental Deterioration” [Title/Abstract] OR “Mental Deteriorations” [Title/Abstract] OR “Cognitive” [Title/Abstract]). Similar combinations of subject headings and free-text terms were applied in other databases. Additionally, consistent with systematic review guidelines, we manually searched for gray literature, including conference proceedings, theses, and government reports, where relevant. The complete search string is provided in Supplementary material.

### 2.3 Inclusion criteria and study selection

We used Populations, Interventions, Comparisons, Outcomes, and Study (PICOS) framework. For inclusion in this review, studies had to meet the following criteria: (1) include older adults (aged ≥60 years) diagnosed with cognitive impairment (including MCI and CF); (2) examine Baduanjin exercise as the sole intervention; (3) include a control group receiving no intervention, usual care, or another therapy; (4) report cognition-related outcomes; and (5) use a randomized controlled trial (RCT) design.

Studies were excluded if: (1) the cognitive impairment of the patients was not clearly defined; (2) the studies involved participants with severe mental or physical conditions that could interfere with the intervention or outcome assessment; (3) Baduanjin exercise was combined with other interventions, making it impossible to assess the individual effects of Baduanjin exercise; (4) the control group engaged in other forms of exercise; (5) outcome indicators were incomplete or missing; or (6) the studies were case reports, case series, review articles, or qualitative studies.

Following the removal of duplicate articles, two reviewers (XG and LW) independently screened all the retrieved records. Initially, the titles and abstracts were assessed to determine whether the full text should be reviewed. If a reviewer found an article ambiguous, the full text was obtained for a detailed evaluation. Finally, both reviewers independently selected articles according to the inclusion and exclusion criteria. Disagreements were resolved through discussion with the corresponding author to reach a consensus.

### 2.4 Methodical quality assessment

Two reviewers (XG and FL) independently evaluated the methodological quality of the RCTs using the Cochrane risk of bias tool (25). The assessment covered the following aspects: (1) random sequence generation (selection bias); (2) allocation concealment (selection bias); (3) blinding of participants and investigators (performance bias); (4) blindness of outcome assessments (detection bias); (5) incomplete outcome data (attrition bias); (6) selective outcome reporting (reporting bias); and (7) other biases. Based on the guidelines provided in the Cochrane Handbook, each domain of the included studies was classified as having a low risk of bias, high risk of bias, or unclear risk of bias. Discrepancies between the two reviewers were resolved through discussions with a third reviewer (AZ).

### 2.5 Data extraction

Two reviewers (LY and FL) independently extracted data. Any disagreements were resolved through discussion and consensus with the corresponding authors. The following information was extracted from the included studies: general information (title, authors, year of publication, and geographical location of the trials); study characteristics, including baseline sample size, age, number of participants in each group; intervention characteristics (frequency and duration of Baduanjin exercise, control conditions); and outcome measures for global cognitive function, executive function, memory function, and physical frailty. For articles with missing data, the corresponding author was contacted via email to request the necessary information.

### 2.6 Data synthesis

RevMan 5.4 software from the Cochrane Collaboration (25) was used to conduct the meta-analysis. To examine the effect of Baduanjin exercise on cognitive impairment in older adults, we performed separate qualitative analyses of various cognitive outcomes. The effect sizes for these outcomes were summarized using the inverse variance method, weighting individual studies accordingly. Depending on the measurement tools used for specific outcomes, we reported either the mean difference (MD) or the standardized mean difference (SMD) along with their 95% confidence intervals (CIs). The statistical heterogeneity among the included studies was assessed using the I^2^ statistic. I^2^ values between 25 and 50%, 50 and 75%, and >75% indicated low, moderate, and high heterogeneity, respectively. A fixed-effects model was employed if the I^2^ value was <50% and the *p*-value was >0.1. Otherwise, we applied a random-effects model.

## 3 Results

### 3.1 Literature search

**Figure 1:**
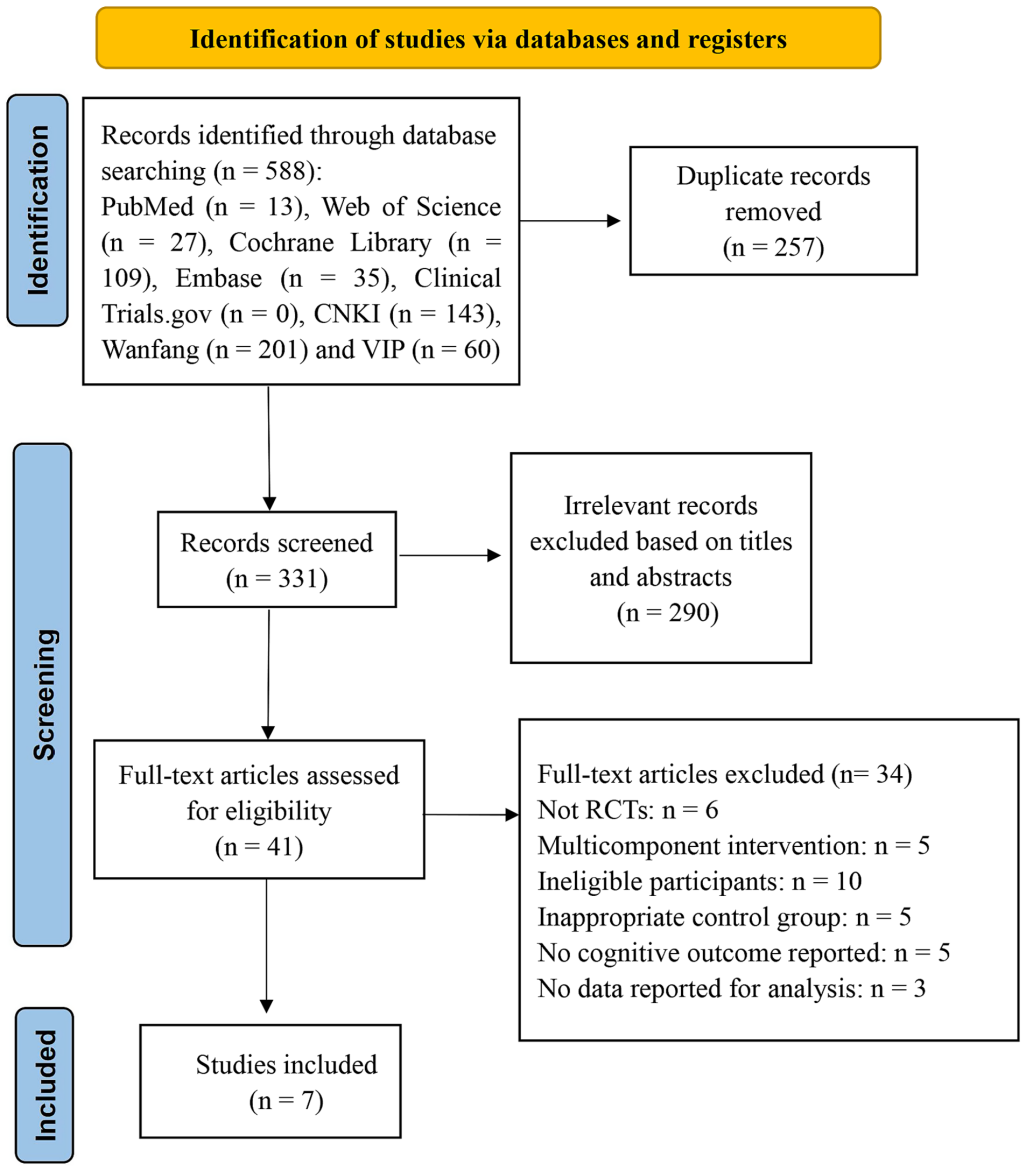
Flow chart of study selection.

A total of 588 potential studies were identified during the initial search, among which 331 were retained after eliminating 257 duplicate articles. After screening titles and abstracts, 290 studies were excluded. We reviewed the full texts of the remaining 41 articles and excluded 34 studies that did not meet the inclusion criteria. The reasons for exclusion were as follows: no RCTs (*n* = 6), multicomponent interventions (*n* = 5), ineligible participants (*n* = 10), inappropriate control groups (*n* = 5), no cognitive outcomes reported (*n* = 5), and no data reported for analysis (*n* = 3). Ultimately, seven RCTs were included in this review (26–32). A comprehensive flowchart depicting the literature screening process is shown in Figure 1.

### 3.2 Study characteristics

Table 1 summarizes the characteristics of the included studies. All seven RCTs were conducted in China, encompassing a total of 539 participants with an average age of 67.1 years (range 65.3 to 71.4 years). Among them, 269 engaged in the Baduanjin exercise, while the remaining 270 formed the control group. The sample sizes of the studies ranged from 40 to 120 participants. The specifics of the Baduanjin exercise protocol differed across studies, with sessions lasting 50–60 min per day, conducted 3–6 times per week, over a period ranging from 12 weeks to 6 months. The control group interventions included health education, usual activities, and blank controls. Outcome measures covered global cognitive functions and specific cognitive domains. All seven studies examined the impact of Baduanjin exercise on global cognitive function using the Montreal Cognitive Assessment (MoCA) scale. Memory function was evaluated in four studies using the wordlist recall test, Auditory Verbal Learning Test, Verbal Fluency Test, and MoCA subscales. Executive function was assessed in four studies using the Clock Drawing Test (CDT), Stroop Test, and MoCA subscales.

**Table 1:** Characteristics of included studies.

Included trials	Sample size (BJ/CG)	Age, mean (SD) (years)	Interventions	Outcome measures
Li 2016 (26)	57 (28/29)	BJ: 66.59 ± 4.02 CG: 65.93 ± 5.13	BJ: 60 min/session, 3 times/week, total 6 months CG: Health education	Global cognitive function: MoCA Memory function: WMS, AVLT
Liu 2018 (27)	60 (30/30)	BJ: 71.23 ± 5.53 CG: 71.60 ± 5.29	BJ: 60 min/session, 6 times/week, total 6 months CG: Blank control	Global cognitive function: MoCA Memory function: Subscales of MoCA Executive function: Subscales of MoCA
Tao 2019 (28)	40 (20/20)	BJ: 66.17 ± 4.17 CG:65.97 ± 5.66	BJ: 60 min/session, 3 times/week, total 6 months CG: Health education	Global cognitive function: MoCA
Wang 2024 (29)	120 (60/60)	BJ: 66.90 ± 4.54 CG: 66.64 ± 5.49	BJ: 60 min/session, 3 times/week, total 24 weeks CG: Blank control	Global cognitive function: MoCA Executive function: CDT, ST Memory function: VFT Physical frailty: EFS
Xia 2017 (30)	90 (45/45)	BJ: 66.16 ± 4.16 CG: 64.41 ± 4.90	BJ: 60 min/session, 3 times/week, total 6 months CG: Health education	Global cognitive function: MoCA Executive function: ST
Xu 2023 (31)	70 (35/35)	BJ: 67.5 ± 7.3 CG: 68.6 ± 7.5	BJ: 50 min/session, 3 times/week, total 12 weeks CG: Health education	Global cognitive function: MoCA Memory function: AVLT
Ye 2024 (32)	102 (51/51)	BJ: 67.68 ± 5.19 CG: 65.35 ± 5.15	BJ: 50 min/session, 3 times/week, total 24 weeks CG: Health education	Global cognitive function: MoCA Physical frailty: EFS

BJ, Baduanjin exercise; CG, control group; MoCA, Montreal Cognitive Assessment Scale; AVLT, auditory verbal learning test; WMS, Wechsler memory scale; CDT, clock drawing test; ST, Stroop test; VFT, verbal fluency test; EFS, Edmonton frailty scale.

Physical frailty was measured in two studies using the Edmonton Frailty Scale. No adverse events related to Baduanjin exercise were reported in these RCTs.

### 3.3 Methodological quality

**Figure 2:**
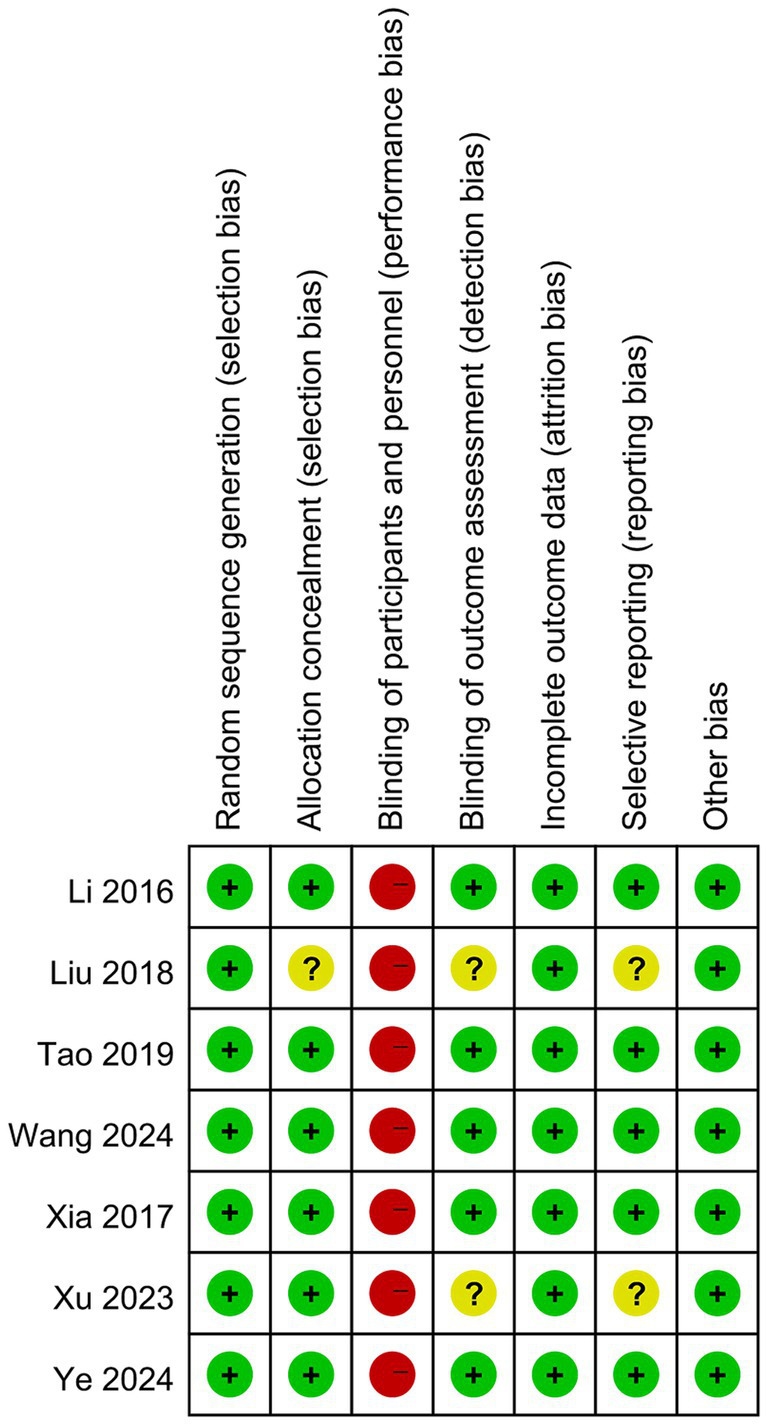
Summary of bias risk for included studies.

Results of the risk of bias assessment for each included study are shown in Figure 2. All the studies implemented random sequence generation (e.g., computer-generated random numbers, random number tables). Six studies (26, 28–32) provided a detailed description of the allocation concealment method (e.g., computer-generated randomization with allocation concealed using opaque sealed envelopes), whereas the remaining one did not specify this information. Given the nature of Baduanjin exercise, where blinding participants and personnel in exercise intervention studies is not feasible, all trials were classified as having a high risk of performance bias. Five trials (26, 28–30, 32) reported that outcome assessors were blinded and were determined to have a low risk of detection bias, while the remaining studies did not report on blinding of the outcome assessors. All seven RCTs provided appropriate descriptions of complete outcome data (e.g., reporting dropout or attrition rates and appropriately handling missing data), resulting in a low risk of attrition bias. Five RCTs (26, 28–30, 32) reported trial registration and were assessed as having a low risk of selective reporting bias, whereas the remaining RCTs were classified as having an unclear risk. All included trials were rated as “low risk” for other biases.

### 3.4 Meta-analysis outcome

#### 3.4.1 Primary outcomes

##### 3.4.1.1 Global cognitive function

**Figure 3:**
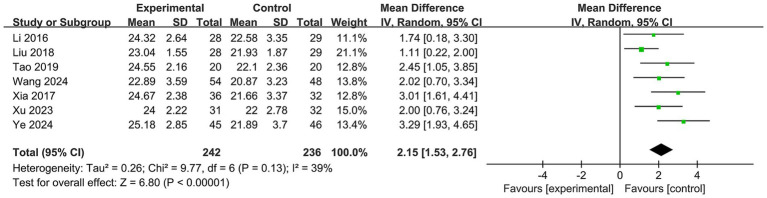
Forest plot of meta-analysis for the efficacy of Baduanjin exercise on global cognitive function.

Seven included studies (26–32) involving 242 participants in the Baduanjin exercise intervention groups and 236 controls assessed global cognitive function using the MoCA (Figure 3). The meta-analysis revealed a significant effect of Baduanjin exercise compared to the control conditions on improving global cognitive function with a large effect size (MD = 2.15; 95% CI, 1.53 to 2.76; *p* < 0.00001). The results showed I^2^ = 39% and *p* = 0.13, indicating a moderate level of heterogeneity.

#### 3.4.2 Secondary outcomes

##### 3.4.2.1 Memory function

**Figure 4:**
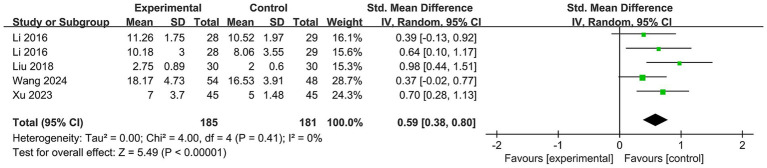
Forest plot of meta-analysis for the effect of Baduanjin exercise on memory function.

We included four studies (26, 27, 29, 31) with 185 participants in the online Baduanjin exercise group and 181 in the control group (Figure 4). The results demonstrated that Baduanjin exercise was more effective than the control in enhancing memory function (SMD = 0.59; 95% CI, 0.38 to 0.80; *p* < 0.00001). No statistically significant heterogeneity was found among the studies (*p* = 0.41, I^2^ = 0%).

##### 3.4.2.2 Executive function

**Figure 5:**
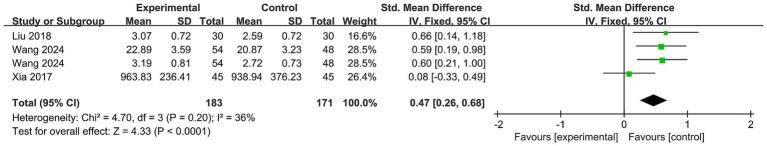
Forest plot of meta-analysis for the effect of Baduanjin exercise on executive function.

Three RCTs (27, 29, 30) reported data on Baduanjin exercise (Figure 5). The meta-analysis indicated a significant effect of Baduanjin exercise on executive function (SMD = 0.26; 95% CI, 0.07 to 0.44; *p* = 0.007) compared to the control condition. However, high statistical heterogeneity was observed (*p* = 0.0002, I^2^ = 82%). After removing the outliers (TMT measure) from Wang’s study (29), the heterogeneity was reduced to a moderate level (*p* = 0.2, I^2^ = 36%), and the improvement in executive function remained significant (*p* < 0.00001).

##### 3.4.2.3 Physical frailty

**Figure 6:**
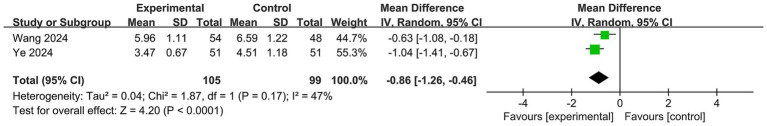
Forest plot of meta-analysis for the effect of Baduanjin exercise on physical frailty.

Two RCTs (29, 32) reported data on physical frailty (Figure 6). The meta-analysis revealed a significantly greater improvement in physical frailty in the Baduanjin exercise group compared to the control condition (MD = −0.86; 95% CI, −0.26 to −0.46; *p* < 0.00001). The results showed I^2^ = 47% and *p* = 0.17, indicating low to moderate heterogeneity.

#### 3.4.3 Adverse effects

No serious adverse events were reported during the exercise training in the included studies. Specifically, three (26, 29, 30) of the seven studies included the intention to monitor adverse events, however, none were ultimately observed. The remaining four studies did not provide information on adverse events.

## 4 Discussion

### 4.1 Summary of evidence

This meta-analysis included seven RCTs with a total of 539 participants to assess the effectiveness of Baduanjin exercise on cognitive impairment in older adults with MCI or CF. The pooled analysis revealed that, relative to control conditions, Baduanjin exercise significantly improved global cognitive function, memory, executive function, and physical frailty. No adverse effects related to Baduanjin exercise were reported in the included studies. These findings suggest that Baduanjin exercise is a safe and effective intervention for improving cognitive impairment in older adults and provides valuable evidence for its potential clinical application.

A previous meta-analysis (22) demonstrated that Baduanjin exercise effectively enhances global cognitive function and memory in middle-aged and older adults. Although these findings are consistent with those of our study, there are some key differences. The study by Wang et al. (22) included a broader population encompassing middle-aged individuals and those with cognitive impairments caused by conditions such as traumatic brain injury, stroke, and chronic obstructive pulmonary disease. Additionally, the control groups in the included studies involved interventions that combined Baduanjin exercise with pharmacological treatments and introduced multiple factors that could influence the outcomes.

Our meta-analysis indicated a significant effect of Baduanjin exercise on improving executive function (*p* = 0.007) compared to the control condition. However, high statistical heterogeneity (I^2^ = 82%) was observed, suggesting that the high heterogeneity observed may be related to variations in the different measurement methods used to assess executive function. Different tools assess executive function from various angles, which could lead to discrepancies in the results. Additionally, differences in the study populations, such as varying baseline cognitive statuses or ages, might also play a role. After removing the outlier (TMT measure) from one study (29), the heterogeneity was reduced to a moderate level (I^2^ = 36%), and the improvement in executive function remained significant (*p* < 0.00001). A previous meta-analysis (33) of 16 RCTs involving 934 patients with MCI found that Baduanjin exercise significantly improved executive function (*p* < 0.05) as measured by the MoCA, CDT, ST, and the Digit Symbol Coding test. While this aligns with our findings, it is important to note that the study by Lin et al. (33) did not limit participants’ age; therefore, it did not specifically focus on older adults.

Additionally, several recent RCTs (29, 32, 33) evaluated the efficacy of Baduanjin exercise in improving physical frailty among older adults with cognitive impairment. The results indicated that, compared to control conditions, Baduanjin exercise significantly improved physical frailty, as measured by the Edmonton Frailty Scale, in community-dwelling older adults with cognitive frailty. However, no previous systematic review or meta-analysis has explored this topic. Our meta-analysis demonstrated that Baduanjin exercise significantly improved physical frailty in older adults with CF, providing evidence-based support for the use of Baduanjin exercise to address physical frailty in older adults with cognitive impairment.

The findings of this systematic review are in agreement with the conclusions of several other reviews (20, 21, 34, 35) that examined how mind–body exercises (e.g., Tai Chi, Qigong, and Yoga) affected global cognitive function, executive function, and memory in patients with cognitive impairment due to various causes. Notably, a recent theoretical review by Lee et al. (36) highlighted the importance of tailoring exercise interventions to specific dementia subtypes (e.g., Alzheimer’s disease, Lewy body dementia), demonstrating that personalized approaches yield superior cognitive outcomes compared to generic exercise programs. The emerging literature supports Baduanjin exercise as a traditional mind–body exercise to improve cognitive impairment in older adults.

### 4.2 Potential mechanisms of Baduanjin exercise

Baduanjin exercise is a mild and safe form of aerobic exercise that incorporates both kinetic and physiological principles. However, unlike other types of aerobic exercise, Baduanjin exercise emphasizes the coordination of mind and body through the integration of breathing, body posture, and focused intention. It primarily aims to regulate practitioners’ mental and psychological states to enhance physiological functions (37). This may explain why Baduanjin exercise offers advantages over general aerobic exercise in some aspects of cognitive impairment interventions. Baduanjin exercise significantly outperforms brisk walking in improving global cognitive function, attention, execution, and processing speed in older adults with mild cognitive impairment (23). However, the exact mechanism through which Baduanjin exercise intervention influences cognitive impairment in older adults remains unclear.

It is now well-established that oxidative stress and inflammation play a crucial role in the development of cognitive impairment in older adults (38–41). Baduanjin exercise can improve physical frailty and cognitive function in older adults with CF and may modulate oxidative stress and inflammatory processes by reducing circulating levels of pro-oxidative markers such as MDA and 8-iso-prostaglandin F2α, while increasing levels of the antioxidant enzyme SOD; additionally, the improvement in cognitive function with Baduanjin exercise is mediated by an increase in circulating inflammatory cytokines, specifically IFN-*γ* and IL-2 (32).

Additionally, practicing Baduanjin exercise can increase Amyloid beta 1–42 peptide (Aβ_1-42_) levels and decrease Tau protein levels in the cerebrospinal fluid (CSF) of MCI patients^42^. This suggests that one mechanism by which Baduanjin exercise improves cognitive impairment may be through delaying Aβ_1-42_ deposition in neurons, regulating Tau levels, and thereby reducing neurotoxicity and protecting neurons (42). Another study found that Baduanjin exercise can regulate the connectivity between the dorsolateral prefrontal cortex and the insula, a brain region that plays a key role in cognitive function, particularly in tasks such as reorienting attention (43).

### 4.3 Strengths and limitations

To the best of our knowledge, this is the first meta-analysis to focus specifically on the effects of Baduanjin exercise intervention on cognitive impairment in older adults. All the studies included in our systematic review and meta-analysis were RCTs, which are recommended as the most appropriate method to evaluate intervention effects (44). The studies included in this meta-analysis involved Baduanjin exercise as a standalone intervention without the combination of any other intervention methods. Similarly, the control groups were either blank controls or provided with general care comparable to the intervention group, without other interventions, such as aerobic exercise or pharmacological treatments. This approach ensures that the final analysis results directly represent the effects of Baduanjin exercise as an independent intervention, making these aggregated data valuable for recommending Baduanjin exercise as an intervention. Additionally, we focused on patients diagnosed with MCI and CF, excluding those with cognitive impairment caused by underlying conditions, such as trauma, cardiovascular and cerebrovascular diseases, respiratory diseases, and Alzheimer’s disease. This reduced the confounding factors and made the target conditions of the intervention more specific, thereby enhancing reliability of the results.

Despite the seemingly positive effects of Baduanjin exercise on cognitive impairment in older adults, it is crucial to recognize certain potential limitations of this review when interpreting its findings. First, this meta-analysis was ultimately based on seven RCTs, including a total of 539 participants, and the relatively small number of included studies and sample sizes is a limitation. Notably, for the secondary outcome, physical frailty was assessed in fewer than 220 participants across only two eligible studies. The limited number of included studies and the small sample sizes may have introduced sampling errors, potentially threatening the validity of the meta-analysis. Second, all the included studies were conducted in China, with no representation from other countries or ethnic groups, which may have led to some bias in the findings and made the conclusions less convincing. Third, despite our efforts to include studies with relatively high methodological quality, methodological risks and other limitations still weaken the strength of the clinical evidence. One major issue is the unavoidable performance bias owing to the difficulty in blinding participants and personnel, which could lead to subjectivity and social desirability bias. Additionally, two of the seven studies did not report whether outcome assessment blinding was used. Given these biases, caution should be exercised when interpreting the results of this systematic review. Fourth, none of the included studies conducted follow-up assessments, making it difficult to predict the long-term effects of the Baduanjin exercise. Finally, only three studies reported adverse events, which also affected our ability to assess the safety of Baduanjin exercise.

### 4.4 Implications for future research

The simplicity and minimal resource requirements of Baduanjin exercise make it particularly suitable for large-scale implementation in community health programs. Compared to pharmacological treatments or facility-based exercise interventions, Baduanjin exercise requires no specialized equipment and can be delivered by trained community health workers at negligible cost. Future research should prioritize economic evaluations and community feasibility studies to facilitate policy adoption. Additionally, to promote its clinical adoption, future research should explore the optimal duration, frequency, and intensity of Baduanjin exercises for patients with cognitive impairment across different ages, underlying conditions, and cognitive levels. This will help refine intervention strategies and safety-monitoring mechanisms, improve patient adherence, and enhance exercise effectiveness and safety.

## 5 Conclusion

This systematic review and meta-analysis demonstrates that Baduanjin exercise significantly improves global cognitive function, memory, executive function, and physical frailty in older adults. Owing to its simplicity, ease of learning, minimal space requirements, and low risk, Baduanjin exercise shows promise for addressing cognitive impairment in older patients and could be recommended for integration into community health initiatives targeting cognitive aging. Nevertheless, several limitations merit consideration. The sample size (n = 539) was relatively small, and all participants were recruited from China, potentially restricting the extrapolation of findings to other populations. Future studies with larger sample sizes and more diverse geographical representation are needed to confirm these results and to determine the broader applicability of Baduanjin exercise across different cultural and demographic groups.

## Data Availability

The original contributions presented in the study are included in the article/Supplementary material, further inquiries can be directed to the corresponding author.
