# Supplementary material for: Effects of Baduanjin exercise on cognitive impairment in older adults: a systematic review and meta-analysis
Source: Front Public Health. 2025 Jul 3;13:1586011. doi: 10.3389/fpubh.2025.1586011 (PMC12267035; doi:10.3389/fpubh.2025.1586011)
Supplement: Supplementary file 1 [file Supplementary_file_1.docx]

**Supplementary Material: search strategy**

**Appendix 1. Pubmed**

(“Baduanjin” [Title/Abstract] OR “Ba duan jin” [Title/Abstract] OR “eight brocade ” [Title/Abstract] OR “eight section brocades” [Title/Abstract])

AND

(“Cognitive Dysfunctions” [Title/Abstract] OR “Cognitive Impairments” [Title/Abstract] OR “Cognitive Impairment” [Title/Abstract] OR “Cognitive Disorder” [Title/Abstract] OR “Cognitive Decline” [Title/Abstract] OR “Mental Deterioration” [Title/Abstract] OR “Mental Deteriorations” [Title/Abstract] OR “Cognitive” [Title/Abstract])

**Limiters**

- Article type: Randomized Controlled Trial

**Appendix 2. Web of Science**

All Fields = “Baduanjin” OR “Ba duan jin” OR “eight brocade” OR “eight section brocades”

AND

All Fields =“Cognitive Dysfunctions” OR “Cognitive Impairments” OR “Cognitive Impairment” OR “Cognitive Disorder” OR “Cognitive Decline” OR “Mental Deterioration” OR “Mental Deteriorations”OR “Cognitive”

**Limiters**

- •Document Types: Clinical Trial

**Appendix 3. Cochrane library**

Title Abstract Keyword = “Baduanjin” OR “Ba duan jin” OR “eight brocade” OR “eight section brocades”

AND

Title Abstract Keyword = “Cognitive Dysfunctions” OR “Cognitive Impairments” OR “Cognitive Impairment” OR “Cognitive Disorder” OR “Cognitive Decline” OR “Mental Deterioration” OR “Mental Deteriorations” OR “Cognitive”

**Limiters**

- Content type: Trials

**Appendix 4. Embase**

All Fields =“Baduanjin” OR “Ba duan jin” OR “eight brocade” OR “eight section brocades”

AND

All Fields =“Cognitive Dysfunctions” OR “Cognitive Impairments” OR “Cognitive Impairment” OR “Cognitive Disorder” OR “Cognitive Decline” OR “Mental Deterioration” OR “Mental Deteriorations” OR “Cognitive”

**Limiters**

- Article type: Randomized Controlled Trial

**Appendix 5. Clinical Trials. gov**

Other terms = “Baduanjin” OR “Ba duan jin” OR “eight brocade” OR “eight section brocades”

**Limiters**

- Study Results: With results

**Appendix 6. CNKI**

Topic= “八段锦”

AND

Topic= “认知”

**Appendix 7. Wanfang**

Topic= “八段锦”

AND

Topic= “认知”

**Appendix 8. Chinese Science and Technique Journal Database (CSTJD/VIP)**

Title Abstract Keyword= “八段锦”

AND

Title Abstract Keyword= “认知”
